# Collateral mutagenesis funnels multiple sources of DNA damage into a ubiquitous mutational signature

**DOI:** 10.1101/2025.08.28.672844

**Published:** 2025-09-01

**Authors:** Natanael Spisak, Marc de Manuel, Molly Przeworski

**Affiliations:** 1 Department of Biological Sciences, Columbia University, New York.; 2 Institute of Evolutionary Biology, Barcelona.; 3 Department of Systems Biology, Columbia University, New York.

## Abstract

Mutations reflect the net effects of myriad types of damage, replication errors, and repair mechanisms, and thus are expected to differ across cell types with distinct exposures to mutagens, division rates, and cellular programs. Yet when mutations in humans are decomposed into a set of “signatures”, one single base substitution signature, SBS5, is present across cell types and tissues, and predominates in post-mitotic neurons as well as male and female germlines [1–3]. The etiology of SBS5 is unknown. By modeling the processes by which mutations arise, we infer that SBS5 is the footprint of errors in DNA synthesis triggered by distinct types of DNA damage. Supporting this hypothesis, we find that SBS5 rates increase with signatures of endogenous and exogenous DNA damage in cancerous and non-cancerous cells and co-vary with repair rates along the genome as expected from model predictions. These analyses indicate that SBS5 captures the output of a “funnel”, through which multiple sources of damage result in a similar mutation spectrum. As we further show, SBS5 mutations arise not only from translesion synthesis but also from DNA repair, suggesting that the signature reflects the occasional, shared use of a polymerase.

Mutations fuel evolution, give rise to heritable disease, drive cancers, and contribute to aging [4–6]. In principle, they can stem from many sources. Point mutations and indels, in particular, can occur at any time in the cell cycle, if lesions caused by endogenous or exogenous damage are repaired erroneously. In addition, such mutations can arise during genome replication, from chance misincorporation of a base opposite a canonical nucleotide or from error-prone replication triggered by unrepaired DNA lesions. Identifying which mutagenic mechanism or damage type predominates *in vivo* in any given cell type or tissue is challenging, all the more so in humans.

One approach is to leverage the fact that these sources contribute differentially across cell types and tissues. Thus, in cancer genomics, it has become common to model single base substitutions (SBS) in a tumor as a combination of a limited number of “mutational signatures”, then infer the signatures from disparate tumor types. In this representation, each mutational signature is a vector of length 96, with an entry defined as a mutation type (e.g., a base pair transition from C:G to T:A) and the identity of the 5’ and 3’ flanking bases. The idea of the approach, as applied to SBS as well as to double base substitutions and indels [7, 8], is that each signature reflects one or a limited number of damage types, deficiencies in repair or other processes that operate to varying degrees across tumors [9, 10]. To date, application to data from the COSMIC database [11] has led to the identification of around 100 signatures, and helped to link mutation profiles to specific exposures: as one example, signature SBS4 to mutagens in tobacco smoke [12, 13]. While the signatures were originally inferred from mutations in tumors, a subset also accounts for mutations in normal somatic cells and the germline [3, 14, 15].

Two of the single base signatures, SBS1 and SBS5, are found across cancers and in nearly all human tissues and cell types [15–17], including in the germline [3, 14, 15], as well as in other mammals [18, 19]. SBS1 is dominated by transitions at methylated CpG sites, and reflects either spontaneous deaminations or errors in DNA replication [20–23]. Modeling suggests that given these sources, SBS1 mutations should track the number of genome replication cycles [3, 21]. Indirect evidence comes from the analysis of cancers, in which the number of SBS1 mutations increases with age of diagnosis [16] and is higher in metastatic tumors relative to their primary tumor counterparts [24]. In non-cancerous tissues, SBS1 mutations predominate in rapidly-dividing cell types [15], but do not increase detectably with age in post-mitotic neurons and contribute little in quiescent cells and the female germline [3, 25]. Such observations have led SBS1 to be described as a “mitotic clock” [26].

Like SBS1, signature SBS5 (Figure 1A) is ubiquitous across cell types. Moreover, it is the main signature in a number of somatic cell types, as well as in male and female germlines [2, 3, 14, 15, 17, 25]. In contrast to SBS1, the number of SBS5 mutations increases with age not only in dividing cells (e.g., glia) but also in post-mitotic cells (e.g., neurons) (Figure 1B-D), and therefore arises in the absence of whole genome DNA replication [2, 3]. The etiology of SBS5 remains unknown, with suggestions that it may reflect a collection of endogenous mutagenic processes [15, 17, 27, 28].

## SBS5 behaves as a single process

**Fig. 1:**
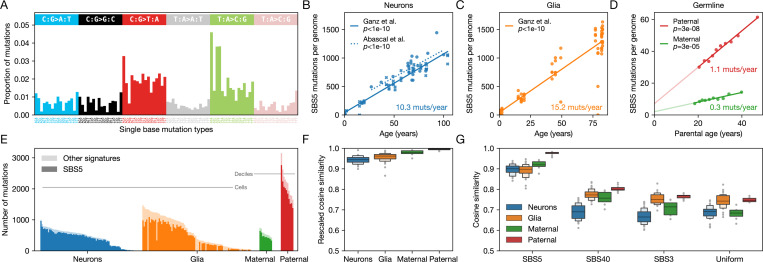
Examples of cell types in which signature SBS5 dominates and provides a good fit to mutation data. (**A**) The distribution of SBS5 mutations over 96 single base mutation types [29]. (**B**) The number of SBS5 mutations increases with age in post-mitotic neurons; data from [25] and [17] are shown with circles and crosses, respectively. Best fit lines and *p*-values are derived from linear mixed-effects models with random intercepts for each individual. Mutation counts per haploid genome (*y*-axis) are adjusted for detection sensitivity [17, 25] (see Methods). (**C**) Same as (B) for glia (data from [25]). (**D**) The number of SBS5 germline mutations increases with deciles of parental age for both maternally- (green) and paternally-phased mutations (red). Data are taken from pedigree sequencing studies and are the subset of mutations phased by transmission in three-generation pedigrees [30, 31]. Best fit lines and *p*-values are from ordinary least squares regression. (**E**) The unadjusted numbers of total mutations (paler shade) and SBS5 mutations (darker shade) detected for individual neuron (blue) and glia (orange) cells, as well as for maternally- (green) and paternally-phased (red) germline mutations (shown in deciles of parental age, as in (D)). Cells and deciles (*x*-axis) are sorted by their total number of mutations (*y*-axis). Data sources are the same as in panels B, C, and D. (**F**) High cosine similarity between observed and reconstructed distributions over 96 substitution types. Shown are the distributions of rescaled cosine similarity. For neurons and glia, we include cells with at least 500 detected mutations. The cosine similarity between the observed and predicted patterns is rescaled by the maximal similarity expected given sampling error (see Methods). Note that the *y*-axis starts at 0.5. (**G**) Cosine similarities after subtracting the contributions of COSMIC signatures other than SBS5 from the observed distributions over 96 SBS types. Residuals are highly similar to SBS5 and differ more markedly from SBS3, SBS40, or a uniform mutation rate over types. Data sources are the same as in panels B, C, and D. Note that the *y*-axis starts at 0.5.

The study of SBS5 presents some technical challenges: for one, its relatively diffuse distribution across possible point mutations (Figure 1A) raises concerns that SBS5 may be hard to distinguish from other “flat” signatures such as SBS3 or SBS40, or that mutations assigned to SBS5 could absorb those that belong to other signatures and thus that the reconstruction may be distorted by leakage from other processes active in the cell [27, 32, 33]. Reassuringly then, SBS5 provides a good fit to cells in which it is the main signature: for instance, on average, it accounts for 62%−86% of mutations in glia, neurons and paternal and maternal germline (Figure 1E; see Methods); the rescaled cosine similarity between the observed mutations and prediction based on COSMIC signature attribution is 0.94−0.99 for these datasets (Figure 1F). After subtracting the inferred contributions of non-SBS5 signatures from the observed distribution over 96 SBS types, the residual distribution of mutations is substantially more similar to SBS5 than it is to other flat signatures or to a uniform rate (Figure 1G; see Methods). Thus, although in some contexts, assignments to SBS5 may be influenced by the presence of other signatures (e.g., SBS16) [26], the inference of SBS5 appears to be robust, given a sufficient number of mutations.

Another challenge in studying SBS5 is interpretative, in that SBS5 could be an amalgamation of distinct endogenous mutational processes that co-occur as a “background” signature (e.g., [27, 33]). Several lines of evidence cast doubt on this possibility, however. First, even across cell types with large numbers of mutations and varying degrees of exposure to mutagens, SBS5 does not decompose into a linear combination of subtypes, in contrast to SBS40 for example [34]. Second, the accumulation of SBS5 mutations with age varies markedly across human cell types (with, e.g., ∼ 0.3 vs ∼ 15 extra SBS5 mutations per year per haploid genome in the maternal germline and glia, respectively; Figure 1C and D). For SBS5 to be the superposition of distinct processes would necessitate them to be highly synchronized in their cell-type-specific age effects. Third, the distribution of SBS5 mutations along the genome is not uniform: for instance, in neurons, SBS5 mutations are enriched in open chromatin and actively transcribed regions, whereas in glial cells, this association is reversed [25]. Such shifts in genomic location would again call for a tight coupling of the various processes underlying SBS5. Therefore, SBS5 behaves as if it were effectively a single process. The question then becomes: what age-dependent process is ubiquitous across diverse cell types?

## Modeling the different modes by which damage leads to mutations

**Fig. 2:**
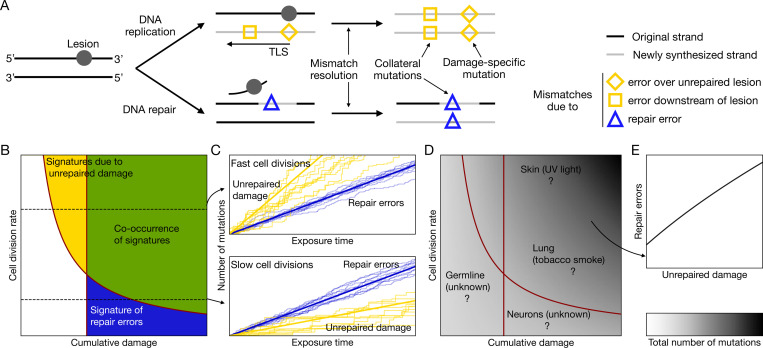
Mutagenesis due to unrepaired and misrepaired damage. (**A**) The cartoon illustrates how a lesion on one of the DNA strands can lead to a mutation at the site of the lesion as well as at a nearby site, thus generating a “collateral mutation”. TLS is triggered by specific lesion types, such as bulky adducts and abasic sites [35]. Repair pathways, such as nucleotide excision repair (NER, pictured), mismatch repair, or homologous recombination, can lead to filling a gap ranging from tens to thousands of bases [36]. (**B**) The type of mutational signatures seen in a cell will depend on the cell division rate and the extent of cumulative damage. Red lines delimit thresholds for signature detection (see Methods). The two types of mutational signatures should co-occur in rapidly dividing lineages with sufficiently high cumulative damage. (**C**) Replication across unrepaired damage is the dominant source of mutation when cell divisions are frequent (top panel) and repair errors when cell divisions are infrequent (bottom panel). The thick lines indicate the expected numbers of mutations due to unrepaired damage (yellow) and repair errors (blue) as a function of the exposure time, which is directly proportional to cumulative damage. Stochastic trajectories simulated from the model are represented by thin lines (see Methods). The blue lines are independent of the cell division rate (see equation 13). (**D**) The total number of mutations per cell expected as a function of cumulative damage and cell division rate. Question marks indicate plausible coordinates of various cell types with known and unknown sources of damage. (**E**) If a single source of damage is sufficiently often left unrepaired by whole genome replication and repair occasionally leads to errors, the numbers of mutations assigned to the two signatures (i.e., the damage-specific signature and the signature of repair errors) should be correlated across cells. Similarly, mutational signatures that arise from the same source of unrepaired damage (i.e., the damage-specific signature and the signature of secondary TLS errors) should be correlated.

Given that SBS5 is seen across disparate contexts, we reasoned that bringing together observations from tumors, non-cancerous soma and germline under a single framework might help to better understand its etiology. To that end, we developed a mathematical model of mutagenesis that considers the efficiency and accuracy of repair and rates of cell divisions (in the model, we assume that whole genome replication cycles are instantaneous and simultaneous with cell divisions) [3, 21]. DNA damage is modeled as occurring in stochastic bursts whose magnitude can temporarily overwhelm the repair capacity of the cell (see Methods). In considering the consequences of DNA damage, we distinguish between two mechanisms by which mutations can arise: replication across or near lesions left unrepaired by the time of whole genome replication, and errors of DNA repair that occur at any other time point (Figure 2A).

Replication across unrepaired DNA damage is error-prone. In some instances, the replicative polymerases can polymerize over the lesion, but the rate of misincorporation is elevated relative to canonical nucleotides: for example, replication over 8-oxo-guanine often leads to a misincorporation of an adenine [37], which contributes to the C:G to A:T substitutions characteristic of oxidative damage and the associated signature SBS18 [38]. For other types of unrepaired DNA damage, such as abasic sites and bulky lesions, the lesion can stall the replicative polymerases and trigger translesion synthesis (TLS), a mechanism of DNA damage tolerance [35]. Because TLS initially recruits low-fidelity inserter polymerases to bypass the lesion, a mismatch can arise directly at the lesion site, represented by a diamond in Figure 2A. For example, TLS over an adduct of benzo[a]pyrene diolepoxide (BPDE) and guanine, a DNA lesion frequently caused by tobacco smoke, leads to the misincorporation of an adenine and contributes to smoking-associated signature SBS4 [13, 39].

DNA damage can also induce mutations at undamaged sites near a lesion, a process that has been termed “collateral mutagenesis” [40–42]. Such errors can arise when TLS polymerases, after bypassing the lesion, introduce a mismatch downstream (represented by a square in Figure 2A), as extender polymerases synthesize DNA for additional base pairs [43]. Alternatively, collateral mutations may arise through polymerase errors during DNA repair synthesis. For instance, during nucleotide excision repair (NER), a polymerase error during gap-filling can lead to a nucleotide misincorporation either downstream or upstream from the lesion site (represented by a triangle in Figure 2A). These mismatches can then be resolved into a (double-stranded, permanent) mutation during subsequent DNA synthesis, through mismatch repair or during genome replication (the “mismatch resolution” step in Figure 2A). Mutations due to error-prone replication across lesion sites will contribute to mutational signatures that reflect the specific type of damage; in what follows, we refer to such signatures as “damage-specific”. In contrast, because collateral mutagenesis occurs at sites removed from the position of the original lesion, it will lead to a signature primarily shaped by the error spectra of the polymerases, rather than by the type of damage.

The model allows us to compare how mutations contributing to damage-specific signatures and signatures of collateral mutagenesis are expected to accrue in different settings. We focus on the regime that is realistic in most *in vivo* contexts, in which the vast majority of lesions are repaired before whole genome replication [44, 45] (see Methods). In this regime, the number of mutations that stem from misincorporations of nucleotides across the lesion or TLS errors downstream from the lesion, denoted $n_{\text{unrepaired}}$, depends on the balance of the frequency f and the mean size of damage bursts b, and the rates of repair $r_{1}$ and $r_{2}$ (which correspond loosely to detection and repair completion, see Methods). This number will accumulate with cell divisions,

$$n_{\text{unrepaired}}\propto \left(\frac{r_{2}}{r_{1}}+b\right)\frac{fb}{Nr_{2}-fb}\phi t,$$

where ϕ denotes the cell division rate, N the number of repair agents, and t denotes exposure time (see Methods). If the cumulative damage, fbt, and the cell division rate, ϕ are high enough, there should be detectable signatures of unrepaired damage (yellow and green areas in Figure 2B). In turn, if virtually all lesions are repaired, but the repair machinery makes errors with non-zero probability ϵ, the mutations due to such errors will arise at a rate that is independent of cell division rates and track cumulative damage; assuming a fixed damage rate, their number will be given by $n_{\text{errors}}\propto \epsilon fbt$. If the cumulative damage is sufficiently high, that is $fbt\gg \epsilon^{-1}$, even non error-prone polymerases used in repair will lead to a substantial number of mismatches and to a detectable signature of repair errors (blue and green areas in Figure 2B).

A key prediction of the model is therefore that in the regime of high cumulative damage and frequent cell divisions, the same source of damage will give rise to two types of mutational signatures, one due to unrepaired lesions and the other to repair errors (green area in Figure 2B). Across cells, the number of mutations contributed by signatures of unrepaired damage should be correlated with one another and with signatures of repair errors (Figure 2E). Moreover, if the same polymerase is occasionally recruited for different damage types and if it synthesizes over neighboring nucleotides that result in a comparable spectrum of misincorporations, then distinct sources of damage will result in a similar signature of collateral mutagenesis. In that sense, and in contrast to the usual interpretation of mutational signatures, collateral mutagenesis will behave as a “funnel”, in which different sources of damage pour into the same mutational output.

## SBS5 tracks multiple sources of DNA damage

We hypothesize that SBS5 is such a funneling signature, explaining its ubiquity across disparate cell types. If so, in cell types exposed to sufficiently high levels of a specific type of damage, the number of SBS5 mutations should increase with the damage-specific signature. With this in mind, we examine the coupling of mutational signatures in whole-genome and whole-exome sequencing of tumors and non-cancerous cells.

**Fig. 3:**
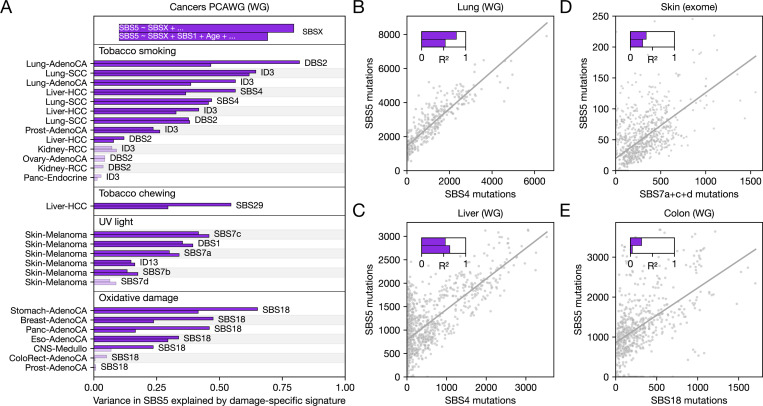
Associations between signature SBS5 and various damage-specific signatures. (**A**) Semipartial $R^{2}$ values quantifying the variance in SBS5 attributed to damage-specific signatures across tumors, with cancer types (left), signatures (right), and etiologies (top) indicated. The upper purple bars show the variance in SBS5 explained by a damage-specific signature in a baseline model; lower bars represent the signature’s contribution to variance after accounting for age, SBS1 (as a proxy for cell division numbers; see main text), and two covariates (ploidy and purity) (see Methods). Solid bars denote comparisons in which the damage-specific association with SBS5 is statistically significant (i.e., *p* < 0.05 after Bonferroni correction). The covariates ploidy and purity are excluded from the top legend for readability (but included in the statistical modeling). (**B**) Association between SBS4 and SBS5 in bronchial epithelial cells in smokers. Mutation counts for each signature were attributed using Signet [46], but similar qualitative conclusions are found using alternative methods (Figure S3). The solid line represents the fit to data from all cells. The upper and lower bars show the semipartial $R^{2}$ quantifying the variance explained by SBS4 in the models, when regressing SBS5 on SBS4 and SBS5 on SBS4, SBS1, and age, respectively. (**C**) Same as (B), but for hepatocytes in smokers. (**D**) Association between SBS7a+c+d (sum of SBS7a, SBS7c and SBS7d) and SBS5 in exomes from microbiopsies of skin. SBS7b was excluded due to its bimodal distribution in this dataset, though its inclusion does not change the qualitative conclusion (Figure S4). The solid line represents the fit to the data from all microbiopsies. Purple bars show the semipartial $R^{2}$ for SBS7a+c+d, as described in (B). Two outlying microbiopsies, which had more than 250 SBS5 mutations, were excluded from the plot to improve visualization (but are included in the statistical analysis). (**E**) Same as (B), but for SBS18 in epithelial cells of the colon. For a comparison of the observed correlation coefficients reported in (B-E) with simulations that assume all signatures are independent, see Figure S2.

In humans, there have been anecdotal reports of associations between the number of SBS5 mutations and damage rates. In lung cancers, the number of SBS5 mutations increases with smoking pack years, a measure of cumulative lifetime tobacco exposure [12]; similarly, in non-cancerous lung epithelia, smokers have a significantly higher SBS5 burden than never smokers [47]. Moreover, in human T6K cell lines, exposures to temozolomide and several platinum-based drugs increase a “background” signature that resembles SBS40 and SBS5 [48, 49]. Using the burden of damage-specific signatures as a proxy for cumulative DNA damage, we tested for broader associations in human mutational data. Analyzing mutations attributed to signatures in the Pan-Cancer Analysis of Whole Genomes (PCAWG [50]), we find that correlations between damage-specific signatures and SBS5 are a general phenomenon. Among 27 well-powered comparisons (upper bars in Figure 3A; see Methods), which include 11 (potentially related) damage-specific signatures and 14 cancer types, 20 are significant associations.

Specifically, we find strong associations between SBS5 and tobacco smoke-related signatures (DBS2, ID3, and SBS4) in lung, liver, and prostate cancers, where these damage-specific signatures account for over half of the SBS5 variance in some cases (Figure 3A). We further detect novel associations between SBS5 and signatures linked to tobacco chewing (SBS29 in liver cancer), UV light exposure (SBS7b/d, DBS1, ID13 in melanomas), and oxidative damage (SBS18 in adenocarcinomas across multiple tissues). To confirm these findings, we use an alternative signature decomposition method that was specifically developed to address the challenges posed by flat signatures such as SBS5 [33]. We recapitulate the correlations with all previously mentioned damage sources, with the exception of tobacco chewing, and uncover additional links to signatures associated with colibactin (ID18) and the chemotherapeutic agent melphalan (SBS99) (Figure S5). These results establish that SBS5 is frequently associated with damage of distinct types.

The correlations between SBS5 and damage-specific signatures could reflect shared age-related processes and/or numbers of cell divisions leading to the tumors, rather than indicating that the same type of damage led to both types of signatures. Since SBS1 serves as a proxy for cell division numbers [3, 16], we assess whether the associations are substantially weakened when including age and SBS1 mutation counts as covariates in our analysis (lower bars in Figure 3A). The number of statistically significant associations and the proportion of SBS5 variance uniquely attributed to damage-specific signatures remain comparable (19 vs 20; Figure 3A). While the associations could reflect other “funneling” processes (e.g., if distinct types of DNA damage cause inflammation that in turn leads to SBS5 mutations), the strengths of the correlations between SBS5 and different damage types in a variety of tumor types support a more direct mechanistic link, as would be expected under collateral mutagenesis (Figure 2E). Also consistent with a causal effect of damage on SBS5, in a mouse model of skin cancer induced by acute exposure to the DNA damaging agent DMBA, the number of SBS5 mutations increases with the DMBA-specific signature [51].

A limitation of bulk sequencing is that it primarily leads to the detection of high-frequency mutations within cell populations. Moreover, while most mutations reported in PCAWG likely predate tumor development [1], it remains uncertain whether associations between damage-specific mutational signatures and SBS5 are also common in non-cancerous cells. We therefore also analyze available non-cancerous datasets where mutations were identified either in single cells [47] or in small clusters of closely related cells [52–54], focusing on single base substitutions, the mutation type that can be most reliably detected in the short-read sequencing data used in these studies. Simulations suggest that with such data, we are able to reliably infer the proportion of SBS5 mutations (see Figure S1).

In people with a history of smoking, we observe a strong association between SBS4 and SBS5 in cells from both the lung (Figure 3B) and the liver (Figure 3C), which persists after controlling for SBS1 mutation counts and age (lower bars in Figure 3B-D). The conclusions remain qualitatively unchanged if we use alternative methods to infer signature contributions (Figure S3; see Methods). Such strong correlations (approximately 65% of the SBS5 variance in lung and and 58% in liver cells) are not seen by chance in simulations (see Figure S2). In principle, inter-individual differences (other than a linear effect of age) could contribute to the observed associations in both tumors (Figure 3A) and non-cancerous cells (Figure 3BC). However, in the non-cancerous datasets, where multiple cells are available per individual, linear mixed-effects models incorporating individual as a random effect confirm that SBS4 remains a highly significant predictor of SBS5 counts (*p* < 10^−10^ in lung and liver). Furthermore, phylogenetic relationships among cells within individuals have little to no impact on the associations (Figure S6). Altogether, these findings indicate that smoking-related DNA damage is the dominant factor driving SBS5 accumulation in lung and liver cells of smokers.

To validate the correlation between the UV light-induced signatures and SBS5 observed in melanomas, we analyze exome sequences from skin microbiopsies [54]. Although the relationship appears noisier than for skin melanomas (Figure 3A), possibly due to the much lower mutation counts in exomes, we again uncover an association between cumulative counts of SBS7a+c+d and SBS5 across microbiopsies after accounting by age and SBS1 counts (Figure 3D). A linear mixed effect model with random effects by individual also supports a significant effect of SBS7a+c+d on SBS5 counts (*p* < 10^−10^), and the association persists after removing the effect of phylogenetic relationships among microbiopsies within individuals (Figure S6). Moreover, we infer a significant contribution of UV light-induced signatures DBS1 and SBS7b to the SBS5 burden in whole genome sequences of 34 single-cell derived skin colonies (Figure S7) [55], as well as in deep bulk sequencing of 74 genes in skin microbiopsies from 11 individuals (Figure S7) [56]. Thus, analyses of mutation data in single cells and small clones corroborate the patterns found in cancer genomes, confirming that the accumulation of SBS5 mutations can arise from distinct damage sources.

Tobacco smoke and UV light primarily lead to bulky lesions, which can trigger collateral mutagenesis due to errors in TLS and/or during NER (Figure 2A). In colonic epithelial cells, mutation counts of SBS5 are correlated with those of SBS18, a signature associated with non-bulky oxidative damage [53]. We also identify this association in the same dataset, using an alternative signature attribution method (Figure 3E, see Methods). The number of SBS18 mutations explains ∼ 25% of the variance in SBS5 across cells (Figure 3E, upper bar in inset), but only ∼ 4% after including age and SBS1 as covariates in the model (Figure 3E, lower bar in inset); a similar drop is observed in small bowel mutations using signature attributions in the original publication (Figure S8) [57]. Therefore, in these two tissues, as distinct from a number of tumors (Figure 3A), the correlation of SBS5 with SBS18 appears mostly due to both signatures tracking cell divisions and, to a lesser extent, age (Figure S9 and Table S1). One possibility is that SBS5 and SBS18 are the outcome of related but not identical types of endogenous damage (e.g., bulky versus non-bulky oxidative damage).

## Clusters of mutations point to errors in TLS

**Fig. 4:**
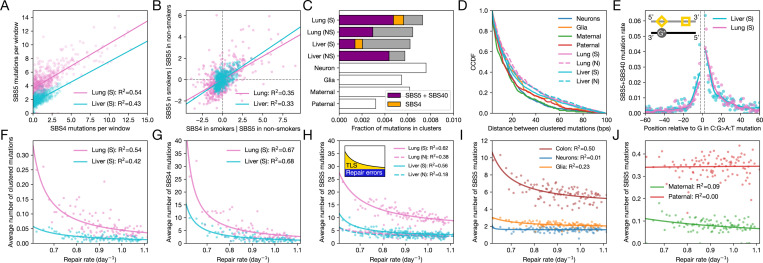
SBS5 is driven by collateral mutagenesis in both TLS and DNA repair. Throughout, for data from lung and liver, smokers are denoted “S” and non-smokers “N”. (**A**) Association between SBS4 and SBS5 mutation rates across 5 Mb genomic windows in lungs and livers of smokers. Signatures were attributed independently in each window, correcting for differences in mutational opportunities (see Methods). The solid line represents the linear regression fit, with the proportion of variance explained shown in the legend. (**B**) Partial correlation between SBS4 and SBS5 across 5 Mb genomic windows in lung and liver of smokers, controlling for the SBS5 rate observed in non-smokers (considered as the baseline; see Methods). The solid line represents the linear regression fit, with the proportion of variance explained shown in the legend. (**C**) Fraction of point mutations in clusters across several human cell types. In the lungs and livers of smokers (“S”) and non-smokers (“N”), the proportion of point mutations in clusters attributed to SBS4 (orange) and SBS5+SBS40 (the sum of SBS5 and SBS40; purple) are shown. Due to low mutation counts, signatures were not attributed to clustered mutations in neurons, glia, and germline; the corresponding distributions over 96 SBS types can be found in Figure S10. (**D**) Distribution of distances between mutations within clusters, excluding mutation pairs separated by < 3 bps (see Methods). CCDF stands for complementary cumulative distribution function (i.e., 1 – CDF). (**E**) Enrichment in the SBS5+SBS40 mutation rate as a function of distance from C:G to A:T mutation in lungs and livers of smokers. To infer their position, we assume that all C:G to A:T mutations result from a damaged guanine (see main text and inset cartoon, where a diamond represents an error across from the G lesion, and a square a secondary TLS error). (**F**) Relationship between rates of NER (x-axis, see Methods) and the average number of clustered mutations (y-axis) across haploid genomes, for lungs and livers of smokers. Each point represents a percentile of the NER repair rate, estimated in 5 Mb windows along the genome. Lines represent the fit of the model $y=\frac{A}{r_{\text{NER}}-r^{*}}+B$, with the proportion of variance explained shown in the legend. (**G**) Same as in (F), but for SBS4 mutation rates. (**H**) Same as in (F), but for SBS5 mutation rates. Inset: Cartoon illustrating the two sources of SBS5 collateral mutations. The total mutation rate (black line) is decomposed into a constant baseline attributed to repair errors (NER) and a TLS component. The colored areas represent the relative contribution of each process across the range of repair rates. (**I, J**) Same as in (F), but for SBS5 mutation rates.

Our analysis shows that signature SBS5 increases with multiple sources of damage across cancers and cell types, in agreement with the funneling hypothesis. We can gain complementary insights by considering the joint distributions of damage-specific signatures and SBS5 along the genome. In smokers, for instance, SBS4 and SBS5 mutation rates are highly correlated across genomic windows (Figure 4A), even after controlling for baseline SBS5 activity in non-smokers (Figure 4B). While the correlation between SBS4 and SBS5 could, in principle, arise from two distinct tobacco-induced lesions (e.g., bulky BPDE lesions bypassed by TLS and another type that leads to to repair errors), the tight association between both signatures across cells and along the genome suggests a single lesion type.

If SBS5 and SBS4 are due to a single lesion type, there are two (non-mutually exclusive) possibilities: they could both originate during TLS triggered by unrepaired damage or SBS5 could result from repair errors. If both signatures result from TLS, with SBS4 reflecting misincorporations at the site of the lesion [13, 39] and SBS5 secondary errors made by TLS polymerases as they continue synthesis, we should detect clusters of point mutations that occur at nearby positions (diamond and square in Figure 2A, respectively) [42, 58]. We find evidence for such events in mutations from the lung and liver of smokers (see Methods), where clusters account for ∼ 0.5% of point mutations, of which SBS4 explains 10 − 12% and SBS5+SBS40 22 − 64% (Figure 4C; here, SBS5 and SBS40 are combined, because it is challenging to distinguish between them reliably when the number of mutations is very small [46]). The spacing between mutations is consistent with the expected length scale of TLS, with an enrichment of pairs separated by < 100 bps (Figure 4D) [40, 42].

Given that a substantial fraction of C:G to A:T mutations within clusters in lung and liver of smokers are attributed to SBS4 (Figure 4C), they likely result from tobacco-induced guanine adducts [13]. We can therefore polarize the clustered mutations with respect to such C:G to A:T mutations and examine where the proximal, putative SBS5 mutation occurs (noting that this polarization will not be perfect, as a subset of SBS5 mutations will also be C:G to A:T substitutions, Figure 1A). As expected if SBS5 mutations in clusters with C:G to A:T mutations arose from TLS errors downstream of the lesion, the positional asymmetry of SBS5+SBS40 mutations is in agreement with the direction of DNA replication (Figure 4E; the two signatures are again combined due to the low number of mutation in clusters). The properties of these mutation clusters support a direct link between SBS5+SBS40 and TLS, consistent with observations of a decreased number of SBS5 mutations in breast cancer cell lines lacking the TLS polymerase REV1 [59] (see also [48]) and a positive correlation between TLS polymerase theta (Polθ) expression levels and SBS5 mutation burden across human cancers [46].

## Evidence for a contribution to SBS5 from errors in repair

TLS cannot fully explain our findings, however. Firstly, there is a similar proportion of clustered mutations in the genomes of non-smokers, even though the scarcity of damage-specific signatures suggests that error-prone TLS is less common (as expected, SBS4 does not contribute to clusters in lungs and livers of non-smokers (Figure 4C)). Furthermore, there are comparable proportions of clustered mutations and similar distance distributions in cell lineages with low rates of divisions, including post-mitotic neurons, as well as maternal and paternal germlines (Figure 4C-D). These observations establish that mutation clusters can also arise from processes other than TLS operating at a similar length scale. The slight differences in distance distributions among cell types roughly follow their relative cell division rates, possibly reflecting different degrees of reliance on TLS (Figure S11).

Secondly, since only a minute proportion of all mutations occur in clusters (Figure 4C), for TLS to explain the standalone SBS5 mutations would require TLS polymerases to often bypass lesions without making an error but lead to a mutation downstream. Alternatively, the widespread association between SBS5 and damage-specific signatures could arise from collateral mutations owing to repair errors. If we assume that damage and repair rates are largely independent along the genome and that most lesions are repaired (see Methods), our mutational model makes opposing predictions about these two possibilities. If TLS is the dominant source of SBS5, we expect that SBS5 mutation rates should correlate inversely with repair rates (see equation 16 in Methods). If repair errors are instead the primary cause, the number of SBS5 mutations should correlate with rates of damage but not of repair (equation 17 in Methods).

In the lungs and livers of smokers, DNA damage often arises from bulky BPDE-induced lesions, which are primarily repaired by NER [60]. To test our model predictions, we estimated NER rates, $r_{\text{NER}}$, in 5 Mb windows along the genome by fitting the rate of exponential decay of UV-induced lesions over multiple time points, using available data from experiments in a human cell line [61] (see Methods). (We note that damage rates cannot be estimated using existing genome-wide maps of BPDE lesions taken at a single time point, because repair starts removing lesions within cells shortly after BPDE exposure, conflating the signal of damage and repair [13].) Consistent with the prediction for mutations arising via TLS, both the distribution of SBS4 mutations and clusters of mutations containing a C:G to A:T substitution are well approximated by $\frac{A}{r_{\text{NER}}-r^{*}}+B$, where B is the baseline number of mutations and A stands for the contribution of unrepaired damage: 67 − 68% of the variance in SBS4 is explained by estimates of $r_{\text{NER}}$ (Figure 4FG; cf. equation 18 in Methods). Notably, in genomic regions with high repair rates, SBS4 mutations are rare, suggesting that damage-specific mutations contribute little at these loci and the baseline number of mutations B∼0 (Figure 4G).

If most SBS5 mutations also arise during TLS due to collateral mutagenesis, a similar distribution pattern would be expected. While SBS5 mutation rates in smokers are also well approximated by $\frac{A}{r_{\text{NER}}-r^{*}}+B$ (Figure 4H, 56 − 62% of the variance explained), unlike for SBS4 and mutation clusters, SBS5 shows a substantial estimate of B, even in the regions with highest repair rates. This observation points to a fraction of SBS5 mutations that arise from errors in repair (Figure 4H inset).

Where TLS is rare, SBS5 should depend much less on repair rates along the genome (17). Accordingly, in non-smokers, there is a much weaker relationship between SBS5 and $r_{\text{NER}}$ ($R^{2}=18-38\text{\%}$; Figure 4H). Similarly, in neurons, in which the vast majority of mutations arose in the post-mitotic state [2], the relationship with repair rates is very weak ($R^{2}=1\text{\%}$; Figure 4I, blue); a weak dependence on repair rates is also observed in maternal germline mutations and no dependence in the paternal germline ($R^{2}=0-9\text{\%}$; Figure 4J, green and red). In turn, a modest relationship is found in glia ($R^{2}=23\text{\%}$; Figure 4I, orange), and a much stronger one in the more rapidly dividing colonic epithelial cells ($R^{2}=50\text{\%}$; Figure 4I, brown). These observations are consistent with predictions of our model, under which the relative contribution of TLS versus repair errors should depend on how often unrepaired lesions persist until genome replication: all else being equal, in cell types with low cumulative damage or infrequent cell division, such as neurons and the germline, SBS5 mutations driven by repair errors are expected to play a more important role (Figure 2C). Under the model, the observed patterns in the lungs and livers of smokers suggest that TLS and repair errors each contributes roughly half of SBS5 mutations (Figure 4H, solid lines). In non-smokers, repair errors become the predominant source instead (Figure 4H, dashed lines).

## Discussion

Taken together, our findings indicate that SBS5 is due to collateral mutagenesis originating from two mechanisms, TLS during whole genome replication and repair errors arising at any time point. Among the cell types analyzed, SBS5 mutations arise through both mechanisms in lung, liver, colonic and glial cells, whereas repair errors appear predominant in neurons and the germline of both sexes. That both mechanisms lead to a single signature indicates the two occasionally recruit the same polymerase, which funnels disparate lesions into SBS5 mutations after mismatch resolution (Figure 2A). If repair and tolerance mechanisms recruit this funneling polymerase at different frequencies, a loss of function in a given pathway may either increase or decrease the number of SBS5 mutations, depending on the alternative pathways employed. Differential reliance on the polymerase among pathways may help to explain why, e.g., in urothelial tumors carrying missense mutations in *ERCC2*, a key component of NER, the number of SBS5 mutations increases [62], while in breast cancer cell lines lacking the TLS polymerase REV1, it decreases [59]. Similarly, the number of SBS5 mutations is elevated in mouse cancer models induced by DMBA [51] and other carcinogens [63], but not with DEN [64], and in some mutagen-exposed cell lines [48] but not others [39, 40, 65]. These contrasts suggest that the recruitment of the funneling polymerase depends on the primary and alternative pathways active in a given cell type and the type of damage. Overall, however, at least *in vivo*, the polymerase is relied on enough to generate a ubiquitous mutational signature.

Polymerase sharing among pathways could be an evolutionary adaptation to damage tolerance or may result incidentally, from polymerase kinetics and availability. Error-prone polymerases such as lambda (Polλ) and kappa (Polκ) are possible candidates, given their known roles in both TLS and gap-filling of long-patch BER and NER [35, 66]. Polymerase zeta (Polζ) is also a potential candidate, as it appears to account for a substantial fraction of spontaneous mutations in a human cell line [48] and has been implicated in collateral mutagenesis induced by TLS [67], as well as in the repair of strand breaks [68]. Given the relatively flat and unspecific spectrum of SBS5 (Figure 1A), together with its weak strand asymmetry [29], we would predict that the funneling polymerase has a diffuse error profile, as is known to be the case for an error-prone polymerase (Polϵ) with a mutated proofreading domain [23].

Regardless, our finding that SBS5 reflects a funneling step, in which many types of damage result in the same mutational output, explains its ubiquity across cellular contexts and its “clock-like behavior” [15, 16], i.e., the increase with age. Our results also highlight the difficulty of teasing apart the consequences of exogenous and endogenous sources of damage. An important implication, notably for cancer etiology, is that the burden of mutagens is likely greater than indicated solely by damage-specific signatures.

## Methods

### Model of DNA damage

We previously developed a mathematical model of mutagenesis, which includes chance errors made during whole genome replication as well as mutations that arise from damage left unrepaired or repaired incorrectly at any time point [3, 21]. Here, we extend this model in order to focus on two additional and important features of damage and repair: that it has limited resources and that DNA damage occurs stochastically. By doing so, we are able to ask what happens when repair is efficient relative to the typical cumulative damage accrued per cell cycle, as is believed to be the case in most *in vivo* settings [44, 45], but is occasionally overwhelmed by large bursts of damage, and to formulate testable predictions about the behaviors of unrepaired lesions versus repair errors.

In the following, we describe the interplay of damage and repair within a cell and derive how the number of lesions is expected to increase with time. We then calculate the expected number of mutations that will result in a lineage of dividing cells. Table 1 provides definitions for our notation.

#### Interplay of damage and repair

We consider a source of damage that creates lesions, described by a stochastic process u(t). To account for the limited resources of the repair machinery, we consider that repair is performed by a finite number of ”agents”, denoted N, which engage with lesions at rate $r_{1}$ (”detection rate”) and disengage when repair is completed, at rate $r_{2}$ (”repair rate”). This parsimonious description obviously oversimplifies the multi-step nature of repair pathways such as NER or BER, in which many distinct DNA repair enzymes participate in repair, but captures relevant dynamics for our purposes. The effective parameter N can be thought of as the bottleneck in the repair process (e.g., the number of enzymes required in the rate-limiting step).

The kinetics of the number of undetected lesions x is given by

(1) $$\dot{x}=u(t)-r_{1}xz,$$

where z denotes the number of repair agents available, and we use the dot notation for the time derivative. This number is equal to N if no damage is under repair, and decreases when repair agents engage with lesions, z∈[0,N]. Denoting by $r_{2}$ the rate at which repair is completed (in other words, that the expected time for repair to complete after damage is detected is $r_{2}^{-1}$), this dynamic can be described as

(2) $$\dot{z}=-r_{1}xz+r_{2}(N-z).$$

Throughout, we assume that $r_{2}\gg r_{1}$ or, equivalently, that we can neglect the few lesions that have been detected but for which repair is not yet completed at the moment of whole genome replication. Under this assumption, the number of unrepaired lesions is simply given by x.

We further consider that repair can lead to a mismatch due to a polymerase error. Denoting the probability of such an error by ϵ, the dynamics of the number of repair errors y are given by

(3) $$\dot{y}=r_{2}(N-z)\epsilon .$$

We search for a solution of the model at long timescales, i.e. $t\gg r_{1}^{-1}$, $r_{2}^{-1}$, when fluctuations in the number of available repair agents z can be neglected, and we use a simplifying assumption that z immediately adapts to the current damage load, leading to a quasi-steady state

(4) $$z(x)=\frac{r_{2}}{r_{1}x+r_{2}}N$$

and

$$\dot{x}=u(t)-N\frac{r_{1}r_{2}x}{r_{1}x+r_{2}},$$

$$\dot{y}=\epsilon N\frac{r_{1}r_{2}x}{r_{1}x+r_{2}}.$$

The crucial timescale is set by the harmonic mean of the rate at which lesions engage repair agents, $r_{1}x$, and the rate at which repair is completed, $r_{2}$. Introducing the effective repair rate

(5) $$r(x)=N{\left(\frac{1}{r_{1}x}+\frac{1}{r_{2}}\right)}^{-1}$$

we can now write more simply

(6) $$\dot{x}=u(t)-r(x),$$

(7) $$\dot{y}=\epsilon r(x).$$

The stochastic dynamics described by this set of equations lead to two distinct regimes at long times. First, the regime of efficient repair, in which the repair capacity is greater than the mean damage rate over time, i.e., $Nr_{2}>\bar{u}=\int_{0}^{T}dt\;u(t)/T$. In this regime, the number of lesions approaches a steady state, reflecting the balance of damage and repair. Alternatively, in the regime of overwhelmed repair, the damage rate surpasses the repair capacity, i.e., $Nr_{2}<\bar{u}$, and unrepaired lesions accumulate, $\dot{x}>0$.

We will now calculate the expected number of lesions and repair errors in the regime of efficient repair, and discuss the regime of overwhelmed repair in the subsequent section. The steady-state distribution of the number of unrepaired lesions depends on the distribution of the damage process u(t). As seems plausible, we consider a bursty damage process that can momentarily overwhelm the repair machinery. Denote by b the mean size of exponentially distributed bursts, and f the frequency of bursts so that $\bar{u}=fb$. We can write down the master equation for the probability density p(x,t) that the number of lesions is x at time t,

(8) $$\frac{\partial p(x,t)}{\partial t}=\text{damage in bursts}+\text{repair with limited capacity}\;\frac{\partial p(x,t)}{\partial t}=f\int_{0}^{x}dx^{\prime }\left(v\left(x-x^{\prime }\right)-\delta \left(x-x^{\prime }\right)\right)p\left(x^{\prime },t\right)+\frac{\partial }{\partial x}[r(x)p(x,t)]$$

where $v(x)=e^{-x/b}/b$ denotes the distribution of burst sizes. At steady state, the time derivative is equal to zero, and the steady-state distribution p(x) can be found by applying the Laplace transform to the resulting equation (similar master equations arise in the context of gene expression kinetics [69, 70]). The result is

(9) $$p(x)=\frac{1}{Z}\left(\frac{1}{r_{1}x}+\frac{1}{r_{2}}\right)x^{f/Nr_{1}}e^{-\frac{Nr_{2}-fb}{Nr_{2}b}x},$$

a weighted mean of two gamma distributions; Z denotes a normalization constant. The expected number of lesions is given by

(10) $$\langle x\rangle =\left(\frac{r_{2}}{r_{1}}+b\right)\frac{fb}{Nr_{2}-fb}.$$

In the limit of $Nr_{2}\gg \bar{u}$, i.e., when all damage is repaired before the next burst, we recover the result of the simpler model from [3], $\langle x\rangle =\bar{u}/Nr_{1}$.

The expected number of repair errors is given by

(11) $$\langle y(t)\rangle =\epsilon \langle \int_{0}^{t}dt^{\prime }u\left(t^{\prime }\right)-x(t)\rangle ≃\epsilon \langle \int_{0}^{t}dt^{\prime }u\left(t^{\prime }\right)\rangle =\epsilon fbt,$$

where we assume that at long times, $t\gg r_{1,2}$, most lesions have been repaired, $\int_{0}^{t}dt^{\prime }u\left(t^{\prime }\right)\gg x(t)$, and therefore the $\epsilon \langle x(t)\rangle$ term is negligible. As a result, the number of repair errors y(t) is a compound Poisson process that tracks cumulative damage.

#### Accumulation of mutations

In this subsection, we calculate the expected numbers of mutations in lineages of cells dividing at rate ϕ. To this end, we make the simplifying assumption that whole genome DNA replication is instantaneous, and simultaneous with cell division, so that the rate of cell division sets the time to the next whole genome replication. As outlined in the main text, mutations can be caused by unrepaired damage or unresolved repair errors. In the case of unrepaired bulky lesions, the completion of DNA replication requires translesion synthesis (TLS). We denote by p the probability that TLS is error-free (i.e., that TLS polymerase inserts the correct base across from the lesion), by $\epsilon^{\prime }$ the probability of secondary polymerase error during TLS, and by q the probability that such an error is resolved by MMR (this probability may be lower for TLS errors than for the usual errors of replicative polymerases, for which MMR is highly efficient). The average time between cell divisions is $\phi^{-1}$. The number of mutations due to unrepaired damage is then given by

$$n_{\text{unrepaired}}≃\langle x\left(\phi^{-1}\right)\rangle \phi t\times \left[\frac{\left(1-p\right)}{2}+\epsilon^{\prime }\frac{p(1-q)}{2}+\epsilon^{\prime }\frac{\left(1-p)(1-q\right)}{2}\right].$$

The first term in the bracket is for error-prone TLS, the second for error-free TLS with collateral mutation, and the third for error-prone TLS with collateral mutation (which will lead to a cluster of two mutations). The relative contributions of these three scenarios are determined by TLS characteristics $\left(p,q,\epsilon^{\prime }\right)$, and in practice will depend on the type of damage.

To calculate the number of mutations due to repair errors, we assume for simplicity that overall half of the mismatches will lead to mutations. This assumption is sensible, given that outside of whole genome DNA replication, MMR is expected to operate symmetrically on both DNA strands [71]. The mismatches that remain unresolved will lead to a mutation in one of the two daughter cells. The number of mutations due to repair errors is thus given by

$$n_{\text{errors}}≃\langle y\left(\phi^{-1}\right)\rangle \phi t\times \frac{1}{2}.$$

In the regime of efficient repair, the contributions of unrepaired lesions and repair errors to the mutation rate per unit of time are given by

(12) $$\frac{n_{\text{unrepaired}}}{t}=\left(\frac{r_{2}}{r_{1}}+b\right)\frac{fb\phi }{Nr_{2}-fb}\left[\frac{\left(1-p\right)}{2}+\epsilon^{\prime }\frac{p(1-q)}{2}+\epsilon^{\prime }\frac{\left(1-p)(1-q\right)}{2}\right],$$

(13) $$\frac{n_{\text{errors}}}{t}=\frac{\epsilon fb}{2}.$$

The mutation rate due to unrepaired lesions depends on the balance of damage and repair rates, as well as on the cell division rate. In turn, the mutation rate due to repair errors is independent of repair rates and is simply proportional to the mean damage rate. To draw the phase diagram in Figure 2B, we assumed for simplicity that a fixed number of mutations $n^{*}$ is required to detect a signature (irrespective of its distribution over 96 SBS types or the presence of other signatures), and plotted the detection thresholds $n_{\text{unrepaired}}=n^{*}$ and $n_{\text{errors}}=n^{*}$ in red.

Due to the shared dependence on damage rates, the expected numbers of mutations that arise from the two sources should be linearly correlated and the slope of the relationship between the two,

$$\frac{n_{\text{errors}}}{n_{\text{unrepaired}}}∼\frac{\epsilon }{\phi },$$

determined by the ratio of the rate of erroneous repair and the rate of cell division (see Figure 2E).

#### Dynamics under overwhelmed repair

For completeness, we provide the results of the model in the overwhelmed repair regime, i.e. when $Nr_{2}<\bar{u}=fb$. Unlike in the previous case, the number of unrepaired lesions increases with time,

$$\langle x(t)\rangle =\left(fb-Nr_{2}\right)t,$$

while the rate at which repair errors accumulate is determined by the repair rate, and is independent from the damage rate,

$$\langle y(t)\rangle =\epsilon Nr_{2}t,$$

As a consequence, the contribution of repair errors to the total mutation rate will be minimal. If we assume the cell continues to divide despite the accumulation of mutations, the two contributions to the mutation rate will be given by

(14) $$\frac{n_{\text{unrepaired}}}{t}=\left(fb-Nr_{2}\right)\left[\frac{\left(1-p\right)}{2}+\epsilon^{\prime }\frac{p(1-q)}{2}+\epsilon^{\prime }\frac{\left(1-p)(1-q\right)}{2}\right],$$

(15) $$\frac{n_{\text{errors}}}{t}=\epsilon \frac{Nr_{2}}{2},$$

and hence $n_{\text{unrepaired}}\gg n_{\text{errors}}$. *In vivo*, such an uncontrolled accrual of lesions is untenable in healthy tissues and would likely lead to cell death. However, this regime could be relevant for the case of transient or pathological cell lineage behaviors.

#### Simulations

Trajectories of the stochastic dynamics are simulated using the standard Gillespie algorithm, given that the times to the next event (of damage, repair, or cell division) are all exponentially distributed [70, 72]. We used them to confirm the accuracy of our analytic approximation assuming a quasi-steady state (4), and to simulate the dynamics described by the original set of kinetic equations (1,2,3). The code used in the simulations is adapted from [73].

#### Variation in mutation rates along the genome

To interpret the patterns of variation along the genome, quantified in windows w of 5 Mb length, we assume for simplicity that all rates (f, $r_{1}$, $r_{2}$, and trivially ϕ) and error probabilities $\left(\epsilon ,\epsilon^{\prime },p\right)$ are the same across the genome. We model the variation of damage rates by varying the expected number of lesions per burst, b=b(w), across the windows w. Furthermore, we consider variation in the effective repair rate along the genome, modeled by varying the effective number of repair agents across the windows, N=N(w). For the two modes of mutation accumulation, we then have

(16) $$n_{\text{unrepaired}}(w)\propto \frac{r_{2}b(w)+r_{1}b^{2}(w)}{r_{2}N(w)-fb(w)},$$

(17) $$n_{\text{errors}}(w)\propto b(w).$$

For sources of damage repaired by NER, assuming that damage and repair rates vary independently along the genome, estimates of NER repair rates $r_{\text{NER}}(w)$ relate to the distribution of mutations due to unrepaired damage via

(18) $$n_{\text{unrepaired}}(w)\propto \frac{1}{r_{\text{NER}}(w)-r^{*}},$$

where $r^{*}$ is the critical rate, below which the repair is overwhelmed by damage. Importantly, in this setting, the distribution of mutations due to repair errors $n_{\text{errors}}(w)$ is expected to be independent of $r_{\text{NER}}$.

If, contrary to our assumption, the rates of damage and repair are not independent along the genome, at the scale of 5 Mb, the expected dependence of mutation rates on the estimates of repair, $r_{\text{NER}}$, will differ. If damage and repair are positively correlated, the repair errors should be positively correlated with $r_{\text{NER}}$ (17), and in the opposite case, we expect a negative correlation. In neurons, where repair errors should predominate, we do not detect a significant effect of NER rates on SBS5 mutations (Figure 4I, blue line, fit of the model (18) explains $R^{2}=0.01$ of variance). More generally, we do not find a positive relationship between mutation rates and NER rates for any of the cell types under study, including in regions of relatively high repair rates, $r_{\text{NER}}>1$ day^−1^, where repair errors are expected to be predominate (Figure 4F-J).

### Data sets analyzed

Data from tumor samples We obtained mutational signature activities in PCAWG for single base substitutions (SBS), double base substitutions (DBS), and small insertions/deletions (ID), along with clinical metadata (age at diagnosis, tumor ploidy, and purity), from [1]. In addition, we obtained signature attributions in PCAWG using MuSiCal, as provided in [33]. Based on their established etiologies in the COSMIC database (v3.4 [11]), we classified 30 COSMIC signatures as “damage-specific” (Table S2).

#### Data from non-cancerous tissues

For neurons and glia, we used mutation data based on sequencing at single-molecule resolution of neurons in [17] and whole-genome amplification of single neurons and glia in [25]. Because these methods do not yield complete genome coverage, we used a global sensitivity of 30% to adjust the mutation burdens in Figure 1B-D, based on estimates in the original publications [17, 25] (to our knowledge, sample-specific sensitivities are not provided).

For maternal and paternal germline mutations, we used whole genome sequencing of trios from three datasets [30, 31, 74]. For all analysis but the inference of clusters, we only considered trios in which over 90% of mutations were phased to maternal or paternal genome. To infer clusters of mutations (data presented in Figure 4CD), we used all phased germline mutations, irrespective of the fraction phased.

For lung and liver tissues, we used whole-genome mutation data from colonies derived from single lung epithelial cells [47] and laser-capture microdissected liver crypts [52]. To investigate the relationship between the tobacco-associated signature SBS4 and SBS5, our analysis was restricted to individuals with a history of smoking.

For skin, we integrated data types from three studies. We analyzed whole-exome mutation data from skin microbiopsies [54], as well as mutational signature attributions from targeted deep sequencing of 74 genes in skin microbiopsies from 11 individuals [56], and whole genome sequences of 40 single-cell derived skin colonies [55]. Because psoralen exposure induces DNA damage that may contribute to SBS5 accumulation independently of UV radiation, we excluded samples with a known psoralen-associated signature from [54].

For intestinal tissues, we used mutation data from laser-capture micro-dissected colonic crypts [53] and signature attributions from the small bowel [57]. For the colon data, where mutations were mapped to phylogenetic tree branches relating different crypts within an individual, we used the tree topologies provided in the original study to assign mutations back to their respective crypts.

To our knowledge, there is no comparable non-cancerous dataset for the signature linked to tobacco chewing, SBS29 (Figure 3A).

Web links to all data files used are provided in Table S3.

### Attribution of mutational signatures

For PCAWG, we used signature attributions previously inferred using SigProfiler [75] and MuSiCal [33]. For the non-cancer datasets, we relied on attributions provided by the original publications, generated using several tools, including SigProfiler, hierarchical Dirichlet process (HDP [76]), and MutationalPatterns [77] (see Table S3).

In addition, we performed de novo assignment of COSMIC v3.1 signatures using SigProfilerAssignment (default settings) on whole-genome mutation data from lung [47], liver [52], and colon [53]. Given that SBS1 and SBS5 could not be confidently separated in exonic skin microbiopsy data using their original approach [53], we inferred signature attribution for this dataset using SigNet, a method better suited for low mutation burdens [46]. We also applied SigNet with default settings to the aforementioned non-cancerous, whole-genome datasets.

To quantify the goodness of fit of the signature attribution P(s), we computed the cosine similarity between the observed distribution over 95 SBS types, P(z), and one reconstructed from the signatures, $\sum_{s}P(z|s)P(s)$, where P(z∣s) denotes the distribution over the 96 types that define a SBS signature. We do not expect a cosine similarity of 1 because of sampling error, which is a function of the number of mutations. In order to account for sampling error, we defined the maximal expected similarity for any given data set as the mean cosine similarity between the observed distribution P(z) and 1000 bootstrap samples. This quantity provides an upper bound on the goodness of fit using signature attribution. In Figure 1F, we report a rescaled similarity, defined as the ratio of the observed cosine similarity and the maximal expected similarity.

#### Signature attribution along the genome

To attribute mutational signatures along the genome, we aggregated all mutations from a given cell type within a given cohort (e.g., smokers versus non-smokers), and grouped the mutations in 5 Mb genomic windows or percentiles of the repair rate $r_{\text{NER}}$ (see section “Estimation of repair rates” below). We attributed signatures using SigNet [46], with an adjustment for mutational opportunities (different 3-mer context content) for each genomic window or percentile of the repair rate.

### Associations among mutational signatures

To quantify the association between damage-specific signatures and SBS5 mutation counts, we used ordinary least squares (OLS) regression. We quantified the variance explained using the semi-partial coefficient of determination $SR^{2}$, which isolates the proportion of total variance in SBS5 counts uniquely explained by a single predictor. This coefficient was calculated as the difference in the model’s $R^{2}$ before and after the inclusion of the predictor of interest. For all analyses, predictor and response variables were standardized via Z-score normalization prior to model fitting, and statistical significance was assessed using a Bonferroni-corrected *p*-value threshold of 0.05.

In addition, we evaluated partial correlations between SBS4 and SBS5 across 5 Mb genomic windows in lung and liver samples from smokers. To control for the background rate of SBS5, we conditioned on the SBS5 levels observed in non-smokers, which we considered the baseline pattern of SBS5 accumulation in the absence of (direct) smoking-induced damage.

#### Data from tumor samples

For each signature-cancer type combination in the PCAWG cohort, we restricted our analysis to tumors with more than 25 mutations attributed to the damage-specific signature, proceeding only if at least 20 tumors remained after this filtering. We fit two OLS models: a baseline model with the damage-specific signature counts as the predictor for SBS5 counts, controlling for tumor purity and ploidy as inferred in [78], and a full model that added age at diagnosis and SBS1 mutation counts (as a proxy for cell divisions; see main text) as covariates to the baseline model.

#### Data from non-cancerous tissues and cell types

Analyses were restricted to individuals with multiple samples (i.e., cells or microbiopsies), considering only samples containing at least one mutation from the relevant damage-specific signature were included. The variance in SBS5 explained by a damage-specific signature was estimated using two models, analogously to the cancer analysis: a model with only the damage-specific signature as a predictor and a second model that included donor age and SBS1 counts as covariates.

To account for the non-independence of samples from the same individual, we tested for statistical significance using linear mixed-effects models. The damage-specific signature and SBS1 counts were included as fixed effects, with a random intercept for each individual. The *p*-value associated with the fixed-effect coefficient for the damage-specific signature was used to assess significance.

To ensure that observed associations were not driven by the genealogical relationships of cells or micro-biopsies within an individual, we performed a resampling analysis. We generated 5000 replicate datasets by randomly sampling a single cell or microbiopsy from each donor. For each replicate, we recalculated the $SR^{2}$ values using the OLS models described above. This procedure allowed us to assess the robustness of the associations in the absence of intra-individual phylogenetic structure (Figure S6).

#### Estimation of repair rates along the genome

To estimate NER rates along the genome, denoted $r_{\text{NER}}$, we fitted an exponential to genome-wide maps of UV-induced cyclobutane pyrimidine dimers in 5 Mb windows, for six time points in a human cell line experiment [61].

### Inference of mutation clusters

We expect to find point mutations in close proximity if they occurred as a result of a single event that lead to multiple mutations (such as collateral mutagenesis) or if they arose independently and by chance are near one another. The probability of chance co-localization increases with the number of mutations in a sample and is higher in regions of the genome with elevated mutation rates. Under a uniform mutation rate, in a segment of the genome of length l, we expect a triangular distribution of distances r [79],

(19) $$P_{0}(r)=\frac{l-r}{\left(\begin{array}{c} l\\ 2 \end{array}\right)}.$$

In practice, heterogeneity in the mutation rate will increase the extent of chance co-localization. To characterize what we expect from such heterogeneity, we use the distances between point mutations that occur in independent individuals, and thus cannot have arisen from the same event. We compute the distances within a chromosome arm, to avoid a lower sensitivity to call mutations around the centromeres leading to artefactual patterns in the distribution of distances. Henceforth, l denotes the length of a chromosome arm. We exclude mutations at distance r=1, which correspond to double base substitutions (DBS), and r=2 which are often characterized by distinct mutational spectra, possibly due to constraints of sequence context [42], and may be an effect of DBS or consecutive insertions and deletions. In the following, we thus consider r=3,…,l−1.

Across all datasets considered (neurons, glial cells, maternal and paternal germline, lung and liver of smokers and non-smokers), we find relatively good agreement of the distribution of distances between samples with the analytic prediction for a uniform mutation rate (19); see Figure S12A for probability density functions and Figure S12B for cumulative distribution functions for a subsample of chromosome arms (2q, 18q, 18p; chosen to represent different lengths l), plotted for all datasets.

Within a sample, we can model the distribution of distances as a mixture distribution,

(20) $$P(r)=\rho P_{1}(r)+(1-\rho )P_{0}(r),$$

where ρ denotes the (pairwise) prevalence, $P_{0}(r)$ is given by (19) and $P_{1}(r)$ stands for the distribution of distances between co-occurring mutations. We use the analytical prediction for the null distribution, given the empirical distribution is noisy at short distances and the analytic approximation provides a good fit (see Figure S12). We assume a negative binomial form for $P_{1}$, which allows us to fit a wide range of possible distributions to the observed distribution of distances. We find the parameters of the negative binomial distribution and the prevalence ρ, by fitting a CDF function in a log-log graph (log CDF(r) vs logr, Figure S14A), using a Levenberg-Marquardt algorithm (scipy function optimize.curve_fit with default parameters). Estimates of prevalence across cell types are shown in Figure S13. In all cell types, we find co-occurring mutations, ρ>0, and the mixture distribution (20) provides a good fit to the data. From the fit, we can estimate the expected false discovery rate for a given threshold distance r (Figure S14B), i.e., the fraction of mutation pairs misclassified as co-occurring, when they in fact only co-localize by chance,

(21) $$\text{FDR}(r)=\frac{\text{FP}}{\text{FP+TP}}=\frac{(1-\rho )\sum_{r^{\prime }<r}P_{0}\left(r^{\prime }\right)}{\rho \sum_{r^{\prime }<r}P_{1}\left(r^{\prime }\right)+(1-\rho )\sum_{r^{\prime }<r}P_{0}\left(r^{\prime }\right)}.$$

For consistency, we choose the same threshold of r=100 across datasets, and exclude data from chromosome arms with FDR(r=100)>5% (Figure S14C).

## Supplementary Material

Supplement 1

Supplement 2

## Figures and Tables

**Table 1:** Parameters and variables of the model.

Notation	Parameter / variable
u(t)	Stochastic process of damage
$\bar{u}$	Mean damage rate
x	Number of lesions
y	Number of repair errors (mismatches)
z	Number of free repair agents
N	Total number of repair agents
$r_{1}$	Detection rate
$r_{2}$	Repair rate
r	Effective repair rate
ϵ	Probability of repair error
b	Mean size of damage burst
f	Frequency of bursts
ϕ	Cell division rate
p	Probability of error-free TLS
$\epsilon^{\prime }$	Probability of collateral TLS error
q	Probability that TLS collateral errors are repaired

## Data Availability

The sources of the mutation data are listed in Table S3. The code to reproduce our analyses is available on GitHub at https://github.com/n-t-n-el/sbs5.
