# Supplementary material for: Collateral mutagenesis funnels multiple sources of DNA damage into a ubiquitous mutational signature
Source: bioRxiv. 2025 Sep 1:2025.08.28.672844. Preprint. [Version 1] doi: 10.1101/2025.08.28.672844 (PMC12424658; doi:10.1101/2025.08.28.672844)
Supplement: Supplement 2 [file NIHPP2025.08.28.672844v1-supplement-2.pdf]

bioRxiv preprint doi: https://doi.org/10.1101/2025.08.28.672844; this version posted September 1, 2025. The copyright holder for this preprint (which was not certified by peer review) is the author/funder, who has granted bioRxiv a license to display the preprint in perpetuity. It is made available under aCC-BY 4.0 International license.

Supplementary information

Supplementary figures

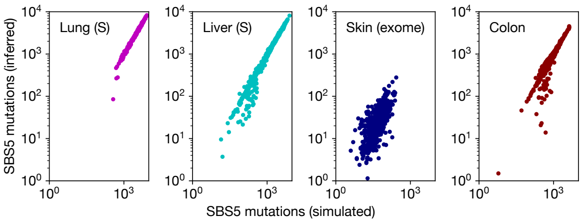

Fig. S1: Test of reliable SBS5 inference across datasets used in Figure 3B-E. We constructed synthetic datasets by shuffling the numbers of SBS5 and damage-specific signatures (as inferred from the data, and reported in Figure 3B-E), obtaining a comparable set of samples with no underlying correlation. We then inferred signature attribution in the synthetic data using SigNet. We report the number of simulated and inferred SBS5 mutations.

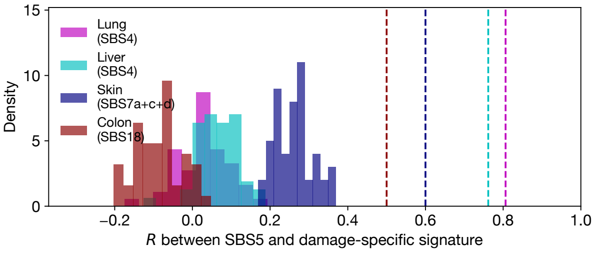

Fig. S2: Testing whether signature attribution can introduces spurious correlations between signatures. We constructed synthetic datasets by shuffling the numbers of SBS5 and damage-specific signatures (as inferred from the data, and reported in Figure 3B-E), obtaining a comparable set of samples with no underlying correlation. We then inferred signature attribution in the synthetic data using SigNet and report here Pearson correlation coefficient between SBS5 and damage specific signatures. For reference, dashed lines denote correlation found in real data (Figure 3B-E), in all four cases significantly greater than the residual correlation found in synthetic data.

bioRxiv preprint doi: https://doi.org/10.1101/2025.08.28.672844; this version posted September 1, 2025. The copyright holder for this preprint (which was not certified by peer review) is the author/funder, who has granted bioRxiv a license to display the preprint in perpetuity. It is made available under aCC-BY 4.0 International license.

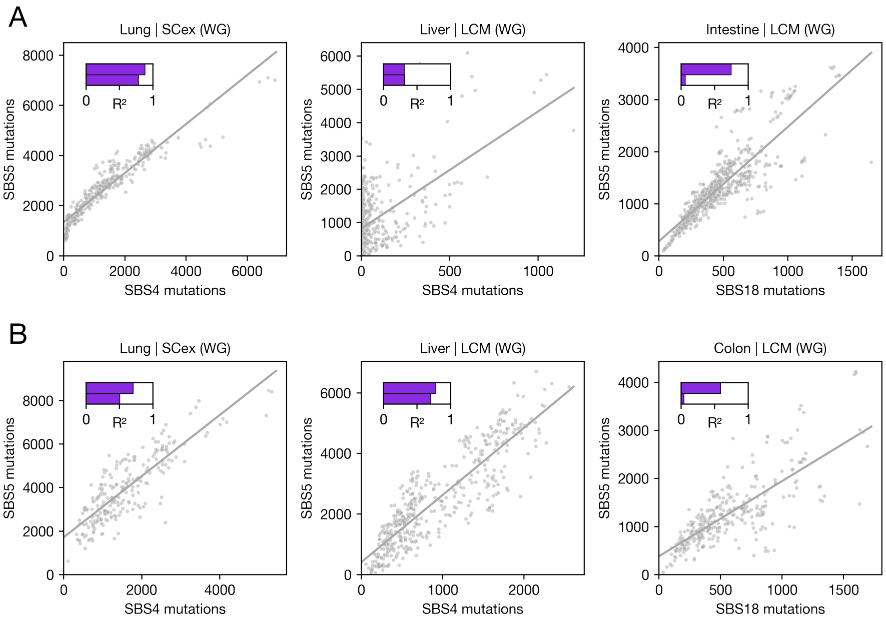

Fig. S3: (A) Association between SBS5 and damage-specific signatures using the signature attributions in the original publications (data sources and signature attribution methods can be found in Table S3). The solid line represents the fit to all data points. The upper and lower bars show the semipartial R^2 quantifying the variance explained by damage-specific signature in the models regressing SBS5 on the damage-specific signature and SBS5 on the damage-specific signature, SBS1, and age, respectively. (B) Same as in (A) but with signature attributions using SigProfiler (see Methods).

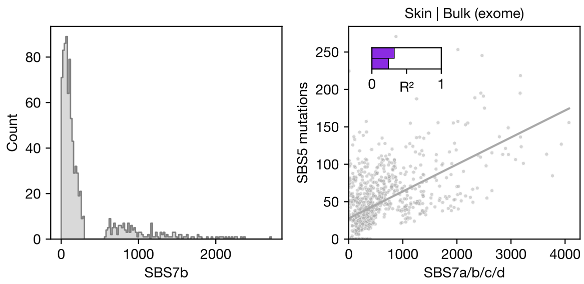

Fig. S4: (A) Distribution of the number of SBS7 mutations attributed by SigNet [46] to skin microbiopsies in ref. [54]. (B) Association between SBS5 and the sum of SBS7a+b+c+d in skin micro-biopsies. The solid line represents the fit to data from all cells. The upper and lower bars show the semipartial R^2 quantifying the variance explained by SBS7a+b+c+d in the models regressing SBS5 on SBS7a+b+c+d and SBS5 on SBS7a+b+c+d, SBS1, and age, respectively.

bioRxiv preprint doi: https://doi.org/10.1101/2025.08.28.672844; this version posted September 1, 2025. The copyright holder for this preprint (which was not certified by peer review) is the author/funder, who has granted bioRxiv a license to display the preprint in perpetuity. It is made available under aCC-BY 4.0 International license.

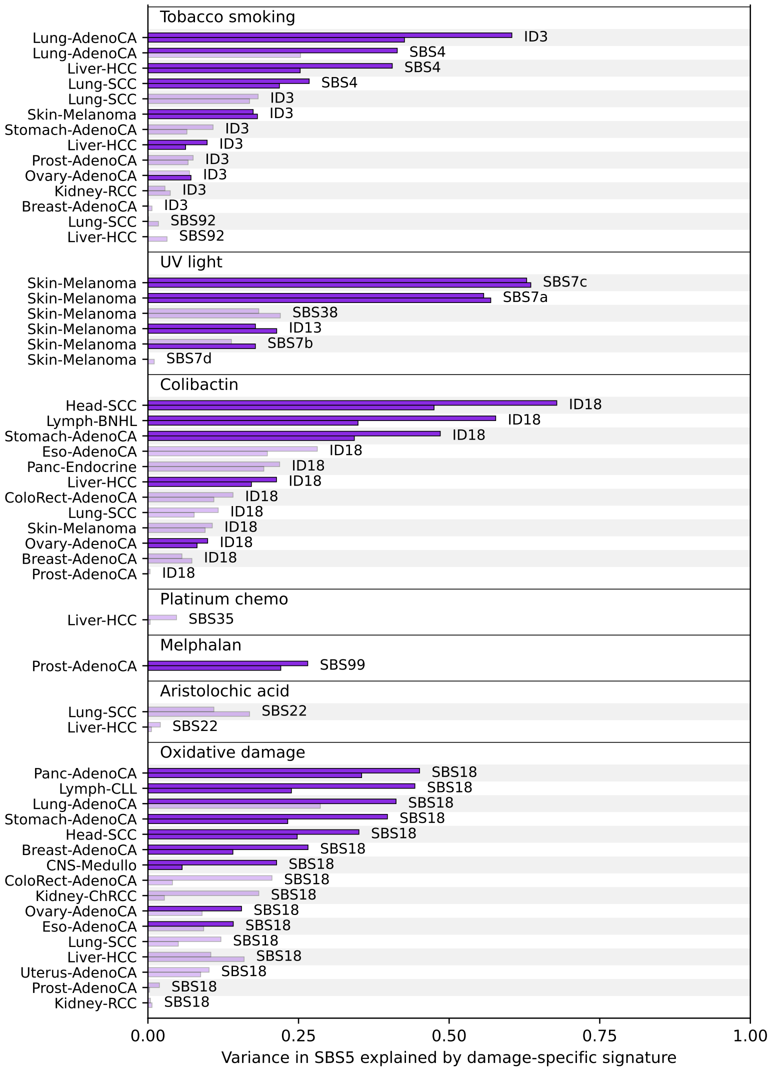

Fig. S5: Association between SBS5 and damage-specific signatures in PCAWG using signature attributions by MuSiCal, as provided in [33]. Semipartial R^2 values quantifying the variance in SBS5 attributed to damage-specific signatures across tumors, with cancer types (left), signatures (right), and etiologies (top) indicated. The upper purple bars show the variance in SBS5 explained by a damage-specific signature in a baseline model; lower bars represent the signature's contribution to variance after accounting for age, SBS1, and two covariates (ploidy and purity) (see Methods). Solid bars denote comparisons in which the damage-specific association with SBS5 was significant ($p < 0.05$ after Bonferroni correction).

bioRxiv preprint doi: https://doi.org/10.1101/2025.08.28.672844; this version posted September 1, 2025. The copyright holder for this preprint (which was not certified by peer review) is the author/funder, who has granted bioRxiv a license to display the preprint in perpetuity. It is made available under aCC-BY 4.0 International license.

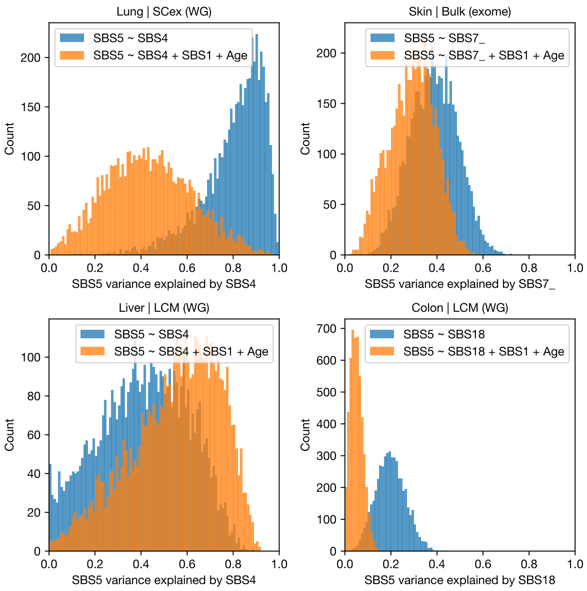

Fig. S6: Distribution of semi-partial R^2 quantifying the variance in SBS5 attributed to damage-specific signatures for 5,000 replicates, each of which resamples a single cell or microbiopsy per individual, for different OLS models (shown in the legend). Datasets are the same as in Figure 3B-E.

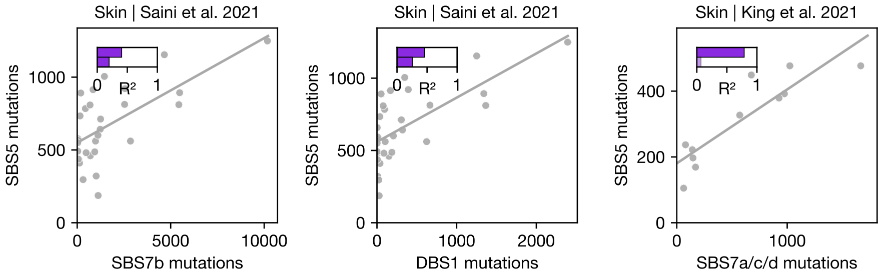

Fig. S7: (A) Association between SBS5 and SBS7b in whole-genome sequences derived from colonies based on single skin cells, using the signature attributions in the original publication [55]. The solid line represents the fit to data from all cells. The upper and lower bars show the semipartial R^2 quantifying the variance explained by SBS7b in the models regressing SBS5 on SBS7b and SBS5 on SBS7b, SBS1, and age, respectively. Solid bars denote comparisons in which the damage-specific association with SBS5 was significant ($p < 0.05$). (B) Same as in (A) but for mutations assigned to the damage-specific signature DBS. (C) Same as in (A) but for mutations assigned to SBS7a+c+d (i.e., the sum of all three signatures) in skin cells sequences from King et al. 2021.

bioRxiv preprint doi: https://doi.org/10.1101/2025.08.28.672844; this version posted September 1, 2025. The copyright holder for this preprint (which was not certified by peer review) is the author/funder, who has granted bioRxiv a license to display the preprint in perpetuity. It is made available under aCC-BY 4.0 International license.

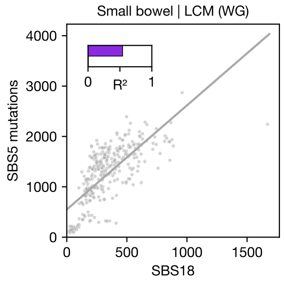

Fig. S8: Association between SBS5 and SBS18 in cells from the small bowel, using the signature attributions in the original publication [57]. The solid line represents the fit to data from all cells. The upper and lower bars show the semipartial R^2 quantifying the variance explained by SBS18 in the models regressing SBS5 on SBS18 and SBS5 on SBS18, SBS1, and age, respectively.

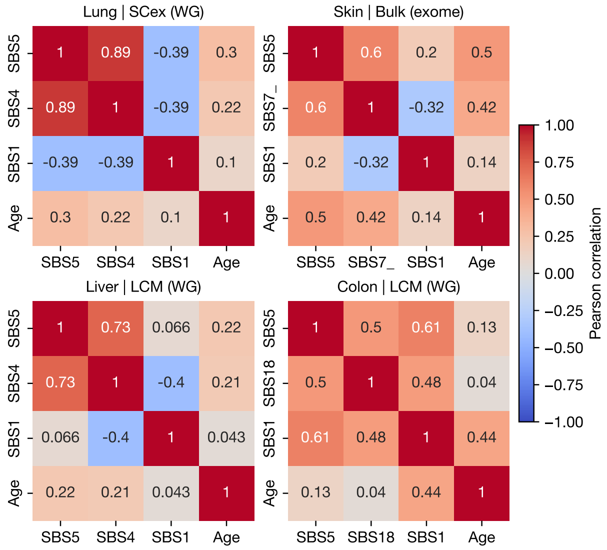

Fig. S9: Pearson correlations between mutation counts assigned to damage-specific signatures, SBS1, SBS5, and age for the data displayed in Figure 3B-E.

bioRxiv preprint doi: https://doi.org/10.1101/2025.08.28.672844; this version posted September 1, 2025. The copyright holder for this preprint (which was not certified by peer review) is the author/funder, who has granted bioRxiv a license to display the preprint in perpetuity. It is made available under aCC-BY 4.0 International license.

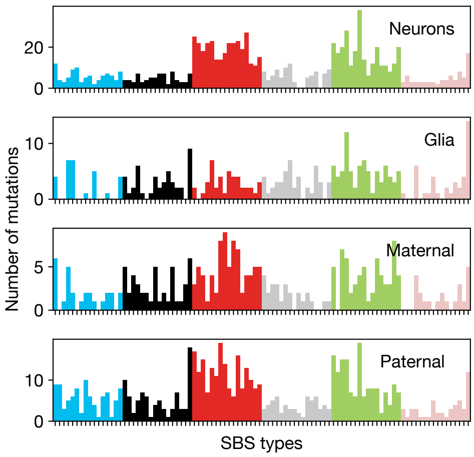

Fig. S10: Distribution over 96 SBS types for clustered mutations in neurons, glia, maternal, and paternal mutations.

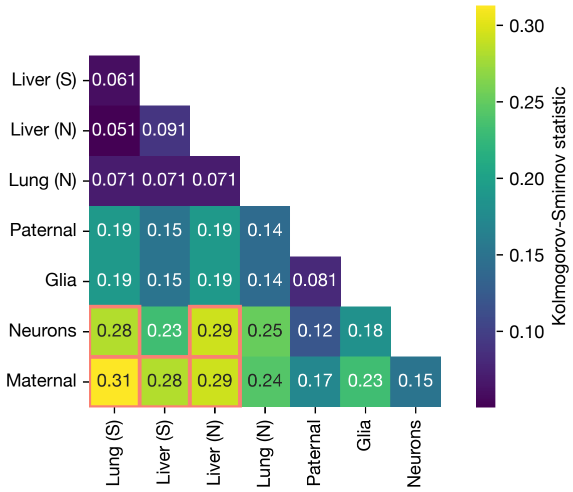

Fig. S11: Kolmogorov-Smirnov statistic for all pairwise comparisons among the CCDFs in Figure 4D. Kolmogorov-Smirnov tests with a p-value below 0.05 (without correction for multiple testing) are indicated with red squares.

bioRxiv preprint doi: https://doi.org/10.1101/2025.08.28.672844; this version posted September 1, 2025. The copyright holder for this preprint (which was not certified by peer review) is the author/funder, who has granted bioRxiv a license to display the preprint in perpetuity. It is made available under aCC-BY 4.0 International license.

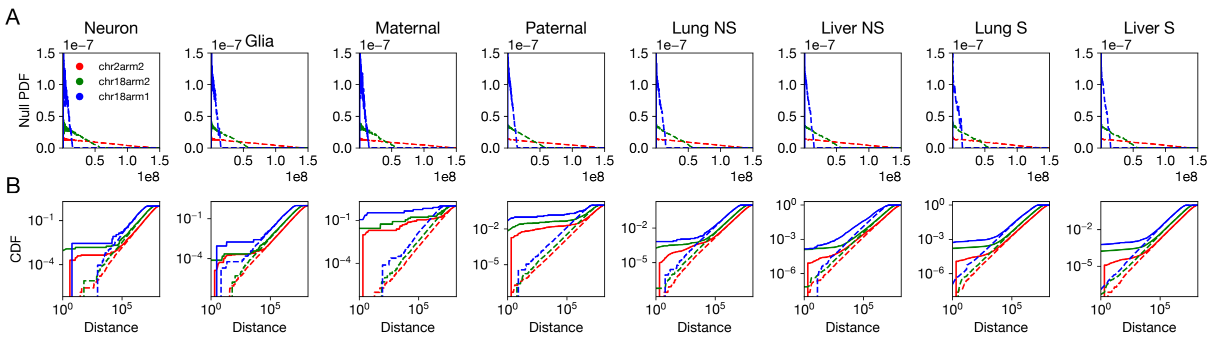

Fig. S12: Distribution of distances for three chromosome arms (2p, 18p, 18q in red, green, blue) across datasets. (A) Probability density of distances between mutations computed between individuals. The distributions agree well with the null expectation (19), a triangular distribution determined by arm length (see Methods). (B) Cumulative distribution function (CDF) of distances between mutations computed within samples (solid lines) and between individuals (dashed line).

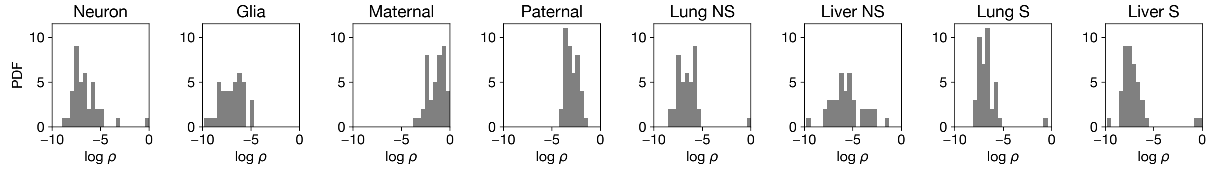

Fig. S13: Distribution of prevalences inferred across chromosome arms for the datasets under study.

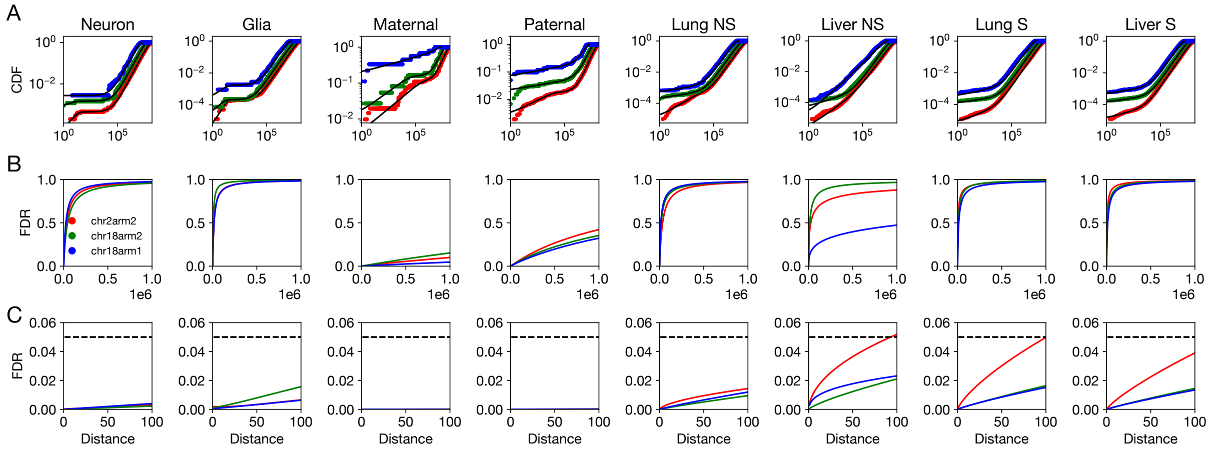

Fig. S14: Modeling the distribution of distances for three chromosome arms (2p, 18p, 18q in red, green, blue) across datasets. (A) The cumulative distribution function (CDF) of distances between mutations computed within samples. Black lines show the best fit of the mixture distribution (20). (B) and (C) False discovery rate estimates for clustered mutations with thresholds at different distances, estimated from the fitted mixture distribution. We impose a threshold of 100 and exclude chromosome arms with FDR> 5% (dashed line in (C)).
